# Decreased overall survival in patients with brain metastases from non-small cell lung cancer with radiotherapy dose on the neurogenic niches

**DOI:** 10.1016/j.tipsro.2026.100380

**Published:** 2026-01-27

**Authors:** Fia Cialdella, Danique E. Bruil, Arthur T.J. van der Boog, Steven H.J Nagtegaal, Filip Y.F.L. de Vos, Joost J.C. Verhoeff, Szabolcs David

**Affiliations:** a Department of Radiation Oncology, University Medical Center Utrecht, Utrecht, the Netherlands; b Department of Medical Oncology, University Medical Center Utrecht, Utrecht, the Netherlands; c Department of Radiation Oncology, Erasmus Medical Center, Rotterdam, the Netherlands; d Department of Radiation Oncology, Amsterdam University Medical Center, Amsterdam, the Netherlands

**Keywords:** Brain metastases (BMs), Non-small cell lung cancer (NSCLC), Radiotherapy, Subventricular zone (SVZ), Hippocampus (HPC), Overall survival (OS)

## Abstract

**Background:**

Non-small cell lung cancer (NSCLC), the most common type of lung cancer frequently leads to brain metastases (BMs), which are associated with a poor prognosis. Radiotherapy is the main treatment for BMs, but irradiates healthy tissue, including neural stem cells (NSCs). NSCs reside in neurogenic niches, mainly the subventricular zone (SVZ) and the subgranular zone (SGZ) within the hippocampus (HPC), which support neurogenesis and brain repair. Radiation damage to NSCs may influence overall survival (OS). Our study aims to evaluate the impact of any radiotherapy dose on NSCs on OS in patients with BMs from NSCLC, with lesion contact explored as a secondary aim.

**Methods:**

We retrospectively included 138 NSCLC patients with BMs and delineated NSC regions on T1 MR-images. Kaplan-Meier (KM) analysis and Cox regression assessed associations between OS, mean radiotherapy dose to SVZ and HPC, and lesion contact.

**Results:**

Higher radiotherapy dose on the SVZ and HPC were significantly associated with reduced OS, with a hazard ratio (HR) of 1.306 ([95% CI 1.043–1.635], p = 0.019) and 1.222 ([95% CI 1.008–1.483], p = 0.041), respectively. Lesion contact with neurogenic niches was also independently significant (SVZ: HR 1.968 [95% CI 1.094–3.542], p = 0.023 and HPC: HR 5.751 [95% CI 1.733–19.07], p = 0.004), although lesion contact was uncommon.

**Conclusion:**

Unintended radiotherapy dose to neurogenic niches is independently associated with worse OS in NSCLC patients with brain metastases, and tumor contact with these regions also shows an adverse association. These findings support considering the SVZ and hippocampus as organs–at–risk in SRS planning and motivate prospective validation in larger, controlled cohorts.

## Highlights

• First atlas-based evaluation of SVZ dose in patients with brain metastases.
• Any RT dose to SVZ and hippocampus independently worsens OS.
• Dose effect persists in SVZ after correction for tumor burden and centrality.
• Tumor contact with neurogenic niches associates with poorer survival.
• Neurogenic niche sparing in SRS may improve survival outcomes.

## Background

### Lung cancer and brain metastases

Most patients with lung cancer are diagnosed with Non-Small Cell Lung Cancer (NSCLC), primarily consisting of adenocarcinoma (LUAD ∼50%) and squamous cell carcinoma (LUSC ∼40%) [1]. A frequent complication of NSCLC is the development of brain metastases (BMs). The incidence of BMs has increased in recent years, primarily driven by improved systemic therapy against the primary tumor that prolongs survival, allowing more patients to develop BMs. Additional contributing factors include more sensitive detection methods and the aging population that is more prone to developing cancers [2]. The exact prevalence of BMs in NSCLC is unknown, with studies reporting a risk rate of 10% to 36% to develop BMs during the disease course [3], [4], [5], [6]. Overall survival (OS) of these patients with BMs varies from median 2 to 9 months depending on type of treatment. Patients with actionable mutations such as EGFR and ALK typically exhibit significantly longer median OS, due to the availability of effective targeted therapies. In contrast, NSCLC patients without these mutations generally show lower median survival rates. This heterogeneity highlights the prognostic importance of molecular profiling in NSCLC BMs [7], [8].

BMs are treated by either local radiotherapy (RT) or a combined treatment of surgical resection and subsequent RT. The aim of local therapy is to improve OS and to alleviate symptoms of BMs [9]. In the Netherlands, the national guideline on brain metastases recommends stereotactic radiosurgery (SRS) as the preferred type of local treatment for patients with up to 10 BMs. This is due to its high precision and lower risk of cognitive side effects compared to whole-brain radiotherapy (WBRT) [10], [11]. Patients with actionable mutations such as EGFR or ALK are generally managed with systemic anticancer therapy as a first-line approach, often before any local intervention [12]. While SRS minimizes exposure to healthy tissue, previous studies on fractionated cranial radiotherapy indicate that RT can still damage healthy brain tissue, leading to atrophy, which may subsequently result in cognitive impairment or other neurological disorders [13], [14], [15], [16]. Radiation to neural stem cells (NSCs) may negatively influence both OS and Quality of Life (QoL), not only through impairment of the brain’s repair capacity but also by contributing to cognitive decline and other neurological disorders that significantly affect patients' daily functioning and wellbeing [17].

### Neurogenic niches in adults

NSCs are located in the so-called *neurogenic niches*, which involve two separate bilateral regions in the adult brain: (1) the subventricular zone (SVZ), situated underneath the ependymal layer of cells that line the ventricles and (2) the subgranular zone (SGZ), part of the dentate gyrus in the hippocampus (HPC) [18]. NSCs induce neurogenesis, which is the process of generating new neurons from NSCs. This process allows cell replacement after neuron loss is triggered by brain injury (e.g. due to tumor growth, surgery or radiotherapy) [19]. However, some experimental drug therapies have been suggested to modulate neurogenesis in preclinical models, systemic anticancer treatments used in NSCLC more commonly cause neurotoxicity and cognitive dysfunction rather than promoting neurogenesis [20]. Adult NSCs, particularly in the HPC, are involved in regulation of several cognitive processes such as learning and memory [21]. Irradiation of the neurogenic niches is hypothesized to reduce adult neurogenesis in two ways: by inducing acute apoptosis in dividing cells and by reducing the production of new neurons through the reduction of the mitotic NSC pool [22]. Preclinical studies in mice support this hypothesis, showing disrupted neurogenesis and associated alterations in the neural microenvironment in response to irradiation of NSCs [18], [23], [24], [25]. These changes have been linked to tumor progression and response to therapy, suggesting a potential link between NSC radiosensitivity and OS. While these studies have primarily focused on the cellular and molecular mechanisms of radiation-induced neurogenesis impairment, their findings offer valuable context for interpreting the effects of radiotherapy on NSCs in clinical settings.

A recent study by our group examined the relationship between tumor contact and unintended irradiation of the neurogenic niches in patients with glioma [26]. We showed that SVZ invasion was associated with decreased survival. No significant association was found for tumors invading the HPC. Additionally, we observed that unintended irradiation of neurogenic areas results in poorer OS in patients with high-grade glioma. These findings suggest that avoiding neurogenic niches during radiotherapy may improve OS outcomes in this patient group.

In contrast, research on neurogenic niches in patients with BMs is limited and predominantly in the preclinical setting. Most clinical studies in BMs focus on the HPC as a potential organ-at-risk, which led to the development of HPC avoidance WBRT (HA-WBRT) [27], [28], [29], [30], [31]. The role of the SVZ in BMs is still largely unexplored, representing a significant gap in the current literature. This contrasts with the focus on the role of the SVZ (and damage to it) in patients with primary brain tumors [32], [33].

As the number of patients with lung cancer BMs is increasing [2], [5] optimizing radiotherapy for this group is critical to extend survival and minimize the negative impact on QoL. Our study investigates the effect of radiotherapy dose on neurogenic niches and its relationship with OS in a cohort of NSCLC patients with BMs. Our primary hypothesis is that any dose on the neurogenic niches is negatively associated with OS in NSCLC patients with BMs, with lesion contact evaluated as a secondary, exploratory factor. Ultimately, our mission is to inform the development of more tailored and effective radiotherapy dose-planning protocols to improve the outcomes for this patient population.

## Materials and methods

### Patient selection and clinical data collection

We retrospectively selected NSCLC patients with BMs, who underwent SRS at a single academic center, the UMC Utrecht (UMCU), between December 2014 and April 2020. All patients were referred by the departments of pulmonology of UMC Utrecht or surrounding general hospitals in the Utrecht region. Inclusion criteria were patients diagnosed with BMs from NSCLC, age ≥18 years and accessible RT planning and dosimetry data. Patients with neuro endocrine pathology and prior intracranial RT were excluded. Patients with a Karnofsky Performance Status (KPS) lower than 70 were only included in the treatment pipeline if neurological improvement as direct effect of SRS was expected.

Clinical data was extracted from the electronic health record (HiX, Chipsoft, The Netherlands) and included demographics (age, gender) as well as clinical information such as type of NSCLC, number of BMs, and prior systemic therapies (i.e. chemotherapy, immunotherapy and molecularly targeted therapy). Type of lung cancer was scored as (1) LUAD, including undifferentiated large cell carcinoma, and (2) LUSC. WHO performance status was derived from KPS using the oncology practice tool [34]. All patients were treated with either single fraction SRS or hypofractionated SRS. Gross tumor volume (GTV) and planning target volume (PTV) were derived from clinical datasets. Date of SRS and date of death were recorded, patients were censored after June 28th 2022. This approach guaranteed a minimum of two-year follow-up period for each participant. The UMC Utrecht Medical Ethics Research Committee approved the study protocol and waived the need for informed consent (METC 18-273).

### Radiotherapy treatment, image processing and segmentation

Patients were treated with 6 MV FFF CT LINAC-based stereotactic radiotherapy (Elekta AB, Stockholm, Sweden) and were immobilized with a thermoplastic head mask (Civco Medical Solutions, Kalona, Iowa, USA) as well as individual head support [35]. The prescribed dose ranged between 15 Gy and 24 Gy depending on GTV size according to the Dutch consensus protocol for brain metastasis radiotherapy. All patients received pre-treatment planning MRIs on a 3 T Philips scanner (Philips Healthcare, Best, The Netherlands) as part of standard clinical care pathway. We collected 3D turbo-spin echo (TSE) T1-weighted MRIs with and without contrast enhancement, with the following parameters: TR = 8.1 ms, TE = 3.7 ms, flip angle = 8°, voxel resolution 1 × 0.96 × 0.96 mm. Simulation CTs were acquired on a Brilliance Big bore scanner (Philips Medical Systems, Best, The Netherlands), with a tube potential of 120 kVp, using a matrix size of 512 × 512 and 0.65 × 0.65 × 3.0 mm voxel size. To obtain a highly conformal dose distribution, volumetric modulated arc therapy (VMAT) using two non-coplanar arcs was employed: one full arc and one partial arc, delivered at couch angles of 0° and 270°, respectively. For patients with multiple BMs an additional couch angle is used at 90°. Treatment planning was performed in the Monaco planning system (Elekta AB, Stockholm, Sweden) [36]. The plan was optimized to ensure that at least 98% of the planning target volume (PTV) received 100% of the prescribed dose, while respecting organ–at–risk constraints and permitting a maximum point dose of up to 130% of the prescription dose within the PTV. MRI to CT rigid registration was performed an in‑house built software (VolumeTool) [37].

**Fig. 1:**
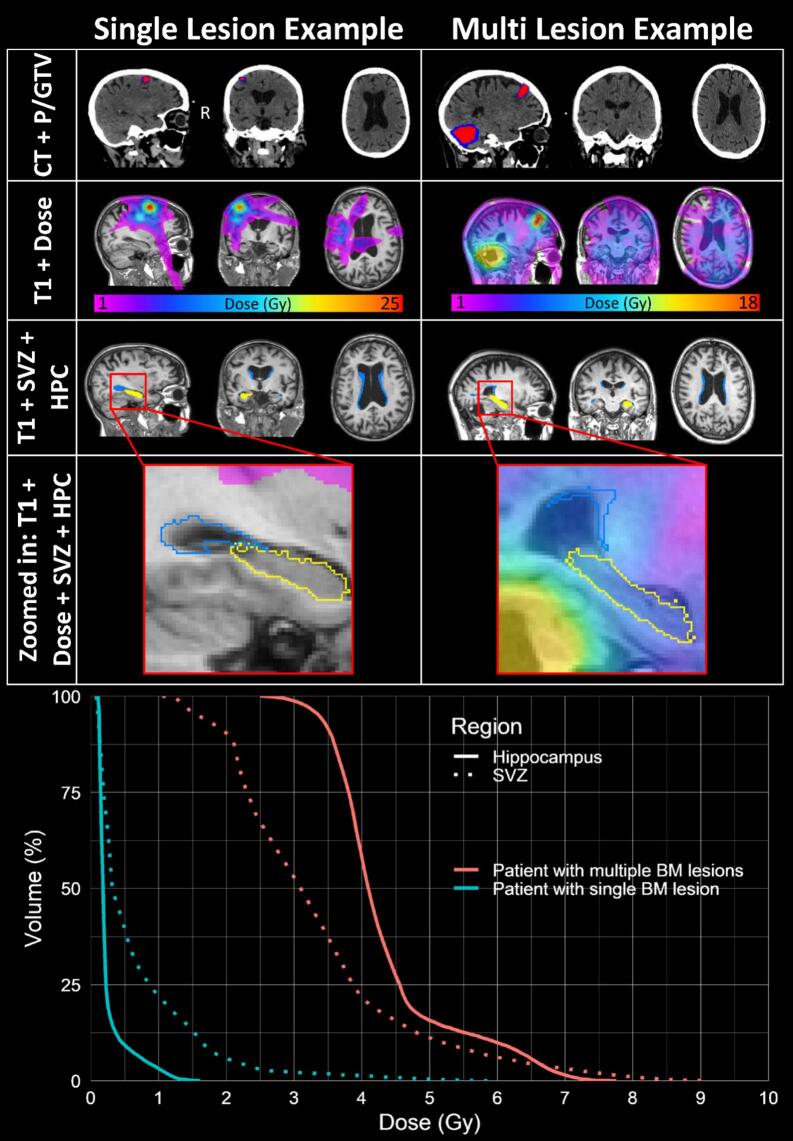
Radiotherapy dose to neurogenic niches in patients with single versus multiple brain metastases. Example of radiotherapy dose to neurogenic niches in a patient with a single brain metastasis (left) and a patient with multiple brain metastases (right). The lower panels show zoomed T1–weighted images with delineated neurogenic niches: the hippocampus (yellow solid contour) and the subventricular zone (SVZ, blue contour) near the metastases. Radiological convention is used (left is right). The bottom graph displays dose–volume histograms for the hippocampus (solid lines) and SVZ (dotted lines); red curves represent the patient with multiple brain metastases and blue curves the patient with a single brain metastasis, illustrating higher dose exposure to neurogenic niches in the multi–lesion case.

First, we created virtual brain grafting (VBG)-enhanced images. When major abnormalities are present in the brain, such as BM lesions, automated segmentation of MRIs with common neuroimaging software, e.g.: FSL, SPM and FreeSurfer, is often challenging and leads to unsatisfactory results. To facilitate the segmentation, we replaced all the abnormal tissues, that is edema, tumor cavity, etc., with healthy appearing, but synthetic tissue [38]. This allowed the automatic delineation of the SVZ and HPC regions on the VBG-enhanced T1 non-enhanced MRIs with standard image processing tools such as the Computational Anatomy Toolbox v12 (CAT12) [39]. The readers are kindly referred to our previous work at Bruil et al. (2022) [26], which describe the fine details of all image processing. In short, images were denoised, segmented and as part of the processing pipeline, all MRIs, and subsequently all related elements, such as masks or dose distribution, were reoriented into a common stereotactic space using the standard MNI template, specifically the MNI152 6th‑generation nonlinear asymmetric version [40], without any resizing of the images. This ensures more direct comparability across subjects, as all brains share the same orientation and aligned coordinate system. In particular, the anterior commissure (AC) is treated as the common central reference point and effectively defines the center of the brain for all patients [41]. An example of the delineation and radiotherapy dose planning is presented in Fig. 1. After the delineation process, the SVZ and HPC regions were segmented into multiple anatomical subregions, each assigned specific labels representing distinct tissue areas within these regions. During the processing of synthetic tissue images (used to model the lesion area), any subregion labels corresponding to synthetic or lesion-affected tissue were excluded from further analysis. Consequently, only the non-affected original parts of SVZ and HPC were included for dose calculations. Mean dose per labeled subregion within the SVZ and HPC were calculated for further analyses. Lesion contact with the SVZ/HPC was defined when SVZ/HPC labels from the segmentation process overlapped with the replaced synthetic tissue. In case SVZ/HPC labels were outside of the synthetic tissue, no lesion contact was considered.

### Dose calculation

Most patients were treated with single‑fraction SRS, while a subset received hypofractionated SRS delivered over multiple fractions. Doses from multi‑fraction regimens were converted to a single fraction equivalent dose (SFED) to ensure consistent biological effect comparison. SFED represents the dose that, if delivered in a single fraction, would yield the same biological effect as the actual multi-fraction regimen. This calculation was performed according to the methodology described by Park [42] and Fowler [43] and recently discussed via Boria [44]. The biologically effective dose (BED) is defined as:

$$\mathrm{BED}=\left(n\times d\right)\times \left(1+\frac{d}{\alpha /\beta }\right)$$

where n is the fraction number, d is the dose and α/β denotes the ratio for early and late responses, which here assumed to be 2, a common value for brain tissue. Note, that BED and dose **d** are not a single value, but rather a 3D map. This equation can be also viewed as:

$$\mathrm{spatialBEDmap}=\left(originaldosemap\right)\times \left(voxelwisecorrectionfactormap\right)$$

If we rearrange the BED equation to find the dose:

$$d^{2}+d\times \alpha /\beta -\frac{\mathrm{BED}\times \alpha /\beta }{n}=0$$

Next, we need to solve this quadratic equation for dose d:

$$d=\frac{-\alpha /\beta \pm \sqrt{{\left(\alpha /\beta \right)}^{2}-\left(4\times 1\times -\frac{\mathrm{BED}\times \alpha /\beta }{n}\right)}}{2\times 1}$$

After simplification and only considering the positive root:

$$d=\sqrt{\frac{{\left(\alpha /\beta \right)}^{2}}{4}+\frac{\mathrm{BED}\times \alpha /\beta }{n}}-\frac{\alpha /\beta }{2}$$

For a single fraction scenario, d = SFED, n = 1; and after substitution of the values:

$$\mathrm{SFED}=\sqrt{1+\left(BED\times 2\right)}-1$$

In practice, the transformation of any (multifraction) dose map into a single-fraction equivalent map goes as follows for arbitrary n and α/β:

1. Divide the multifraction dose map (d x n) with the fraction number (n), to gain access to the voxelwise, per-fraction dose map
2. Divide this per-fraction map with the α/β and add 1. This will result in a voxelwise correction factor map.
3. Multiply the original multifraction map with the voxelwise correction map from #2. This will result in the BED equivalent map of the original multi-fraction map
4. Multiply the BED with 4, divide by α/β, add 1, take the square root, subtract 1 and multiply with $\frac{\alpha /\beta }{2}$
5. The resulting dose map is the SFED equivalent of the original multi-fraction dose distribution

The supplementary materials include a Unix script that converts any dose map into a single fraction equivalent dose. This script requires two input variables: the α/β ratio and the number of fractions in the original treatment plan. To use the script, one must ensure that: (1) the input dose file is in NIfTI format (*.nii or *.nii.gz), and (2) the FSL (fslmaths) tools are installed and accessible.

### Statistical analyses

All statistical analyses were performed using R 2024.09.0 Build 375. The primary outcome of this study was OS, defined as months after the RT until date of death. We utilized Kaplan-Meier (KM) analyses to investigate the impact of lesion contact with the neurogenic niches (SVZ or HPC) on OS. Specifically, we compared patients who had lesion contacting the neurogenic niches to those without contact. Multivariable Cox-regression analyses evaluated associations of both neurogenic niche contact and dose with OS, adjusting for covariates (see Table 1). These included multiple combination of categorical variables (sex, WHO performance status, type of lung cancer, metastases elsewhere in the body, number of BMs, chemotherapy, any other systemic therapy, extent of surgery and contact SVZ or HPC) and continuous variables (age, SVZ, HPC or GTV volume and dose). Multicollinearity was assessed using the Variance Inflation Factor (VIF). Statistical significance was defined at p < 0.05.

**Table 1:** Baseline patient and treatment characteristics.

Patient count (%)	138 (100)
Median age in years (IQR)	67 (60––74)
Sex	
Female (%)	69 (50)
Male (%)	69 (50)
WHO performance status	
0 (%)	30 (21.7)
1 (%)	86 (62.3)
2 (%)	11 (8.0)
3 (%)	4 (2.9)
Unknown (%)	7 (5.1)
Type of lung cancer	
LUAD (%)	102 (73.9)
LUSC (%)	21 (15.2)
Unknown (%)	15 (10.9)
BRAF mutation	
Yes (%)	2 (1.4)
No (%)	40 (29)
Unknown (%)	96 (69.6)
EGFR mutation	
Yes (%)	13 (9.4)
No (%)	47 (34.1)
Unknown (%)	78 (56.5)
ALK mutation	
Yes (%)	3 (2.2)
No (%)	23 (16.7)
Unknown (%)	112 (81.2)
HER2 mutation	
Yes (%)	1 (0.7)
No (%)	32 (23.2)
Missing	105 (78.1)
KRAS mutation	
Yes (%)	22 (15.9)
No (%)	15 (10.9)
Unknown (%)	101 (73.2)
Extracranial metastases	
Yes (%)	45 (32.6)
No (%)	93 (67.4)
Total intracranial volume in cm^3^ (IQR)	1467 (1383–1576)
SVZ volume in cm^3^ (IQR)	10.8 (9.27–13.26)
SGZ volume in cm^3^ (IQR)	1.6 (1.48–1.76)
Number of brain metastases	
1 (%)	71 (51.4)
2–4 (%)	51 (37.0)
>4 (%)	16 (11.6)
Subventricular zone-tumor contact	
Yes (%)	34 (24.6)
Unilateral contact (%)	33 (97.0)
Bilateral contact (%)	1 (3.0)
No (%)	104 (75.4)
Hippocampus-tumor contact	
Yes (%)	5 (3.6)
Unilateral contact (%)	5 (100)
Bilateral contact (%)	0 (0)
No (%)	133 (96.4)
Median single-fraction equivalent dose in Gy (IQR)	21 (21–24)
Median RT dose in SVZ in Gy (IQR)	1.50 (0.87–2.70)
Median RT dose in HPC in Gy (IQR)	1.22 (0.51–2.23)
Radiotherapy scheme	
1 * 15 Gy (%)	3 (2.2)
1 * 16 Gy (%)	2 (1.4)
1 * 18 Gy (%)	18 (13.0)
1 * 20 Gy (%)	3 (2.2)
1 * 21 Gy (%)	44 (31.9)
1 * 24 Gy (%)	44 (31.9)
3 * 8 Gy (%) (SFED = 1 * 14.52 Gy)	23 (16.7)
5 * 6 Gy (%) (SFED = 1 * 14.52 Gy)	1 (0.7)
Median GTV volume in cm^3^ (IQR)	6.40 (2.59–12.89)
Median PTV volume in cm^3^ (IQR)	9.48 (4.10–19.81)
Median distance from PTV CoG to center of brain (in cm, IQR)	6.73 (5.36–7.86)
Brain surgery	
Yes (%)	28 (20.3%)
No (%)	110 (79.7%)
Extent of surgery	
Partial resection (%)	7 (25%)
Complete resection (%)	21 (75%)
Any chemotherapy	
Yes (%)	81 (58.7)
No (%)	54 (39.1)
Unknown (%)	3 (2.2)
Any other systemic therapy	
Yes (%)	67 (48.6)
No (%)	66 (47.8)
Unknown (%)	5 (3.6)

CoG= Center of gravity GTV; Gross tumor volume, LUAD = Lung Adenocarcinoma, LUSC = Lung Squamous Cell Carcinoma, RT = Radiotherapy; SGZ= Subgranular zone, SVZ = Subventricular zone, WHO performance status = World Health Organization performance status.

For exploratory analyses, we additionally examined how PTV size and lesion centrality relate to the dose delivered to the SVZ and HPC. Lesion centrality was approximated by the (Euclidean) distance between the PTV center of gravity (CoG) and the AC, which is commonly treated as the anatomical center of the brain. Centrality distance was only calculated in patients with a single brain metastasis. Although “centrality” is not uniquely defined, we consider this a robust and reproducible measure compared with, for example, the distance to the midline alone. Lesions that are markedly anterior or posterior can lie close to the midline and would then be misclassified as central by a midline-based metric, despite being peripherally located in practice. Using the AC as a central proxy mitigates this problem. Moreover, both the SVZ and HPC are located relatively close to the AC, further supporting the use of AC distance as a biologically and anatomically meaningful centrality measure.

## Results

### Participants

**Fig. 2:**
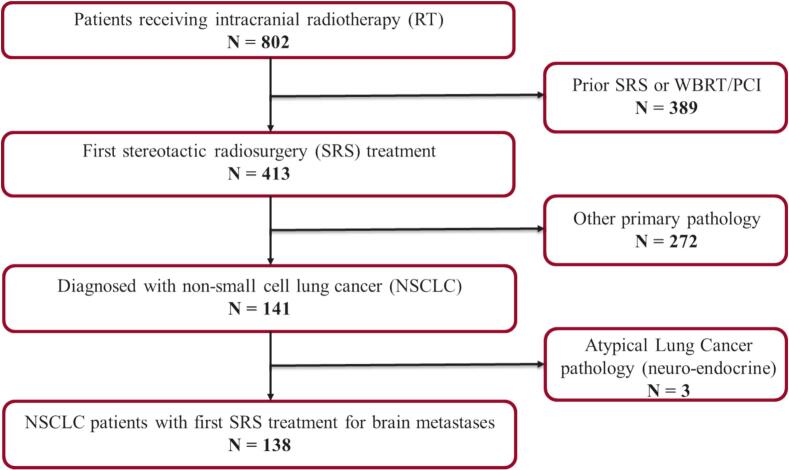
Flowchart of patient inclusion. RT: Radiotherapy, SRS: Stereotactic radiosurgery, WBRT: Whole brain radiotherapy, PCI: Prophylactic cranial irradiation, NSCLC; non-small cell lung cancer.

From December 2014 to April 2020, we identified 413 patients who met the inclusion and exclusion criteria. Of them, 141 were diagnosed with BMs from non-small cell lung cancer (NSCLC). After excluding 3 patients with neuroendocrine pathology, the final study cohort comprised 138 patients. The patient inclusion flowchart is shown in Fig. 2. Baseline patient and treatment characteristics are shown in Table 1**.** The median age was 67 years (interquartile range 60–74) and the male–female distribution was balanced. 24 patients (17.4%) received hypofractionated SRS delivered over multiple fractions. Prior to RT, 28 (20%) patients underwent surgery, of whom 21 patients had a complete macroscopic resection of one or multiple lesions. Regarding systemic treatment, 29.7% of the patients received concurrent chemotherapy with other systemic treatments, 28.4% were previously treated with chemotherapy alone and 19.1% received immunotherapy/targeted therapy as primary regimen. Supplementary Fig. 1 shows the joint distribution of the GTV and PTV values**.** The inverse relationship between tumor size and relative PTV expansion demonstrates that smaller metastases receive disproportionately larger volumes of irradiated normal brain tissue. For tumors <5 cc, PTVs can exceed 200–300% of the GTV, while larger tumors show more modest expansions (120–180%). This size-dependent effect is particularly relevant when treatment margins encompass critical structures such as the HPC or SVZ, as the dose to normal tissue becomes substantially greater relative to tumor volume in small lesions.

### Survival analysis on subventricular zone and hippocampus tumor contact

**Fig. 3:**
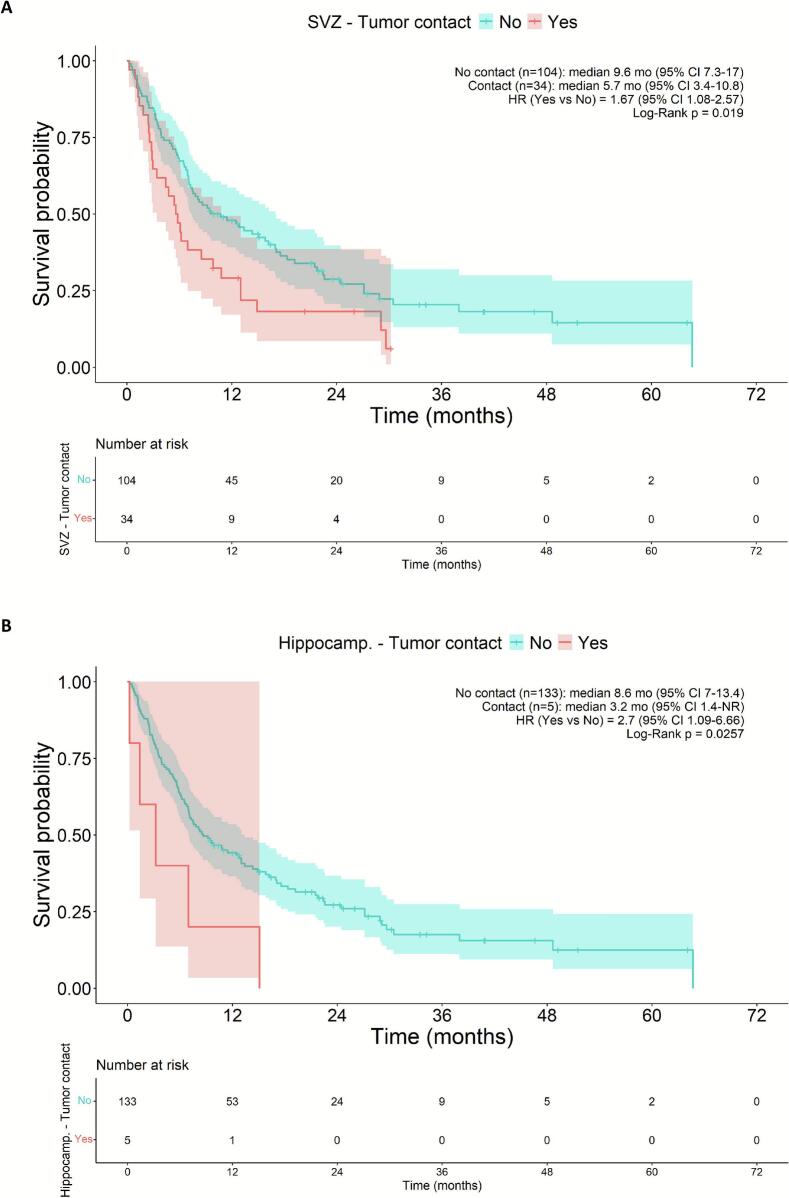
A) Kaplan-Meier overall survival according to tumor contact with the subventricular zone (SVZ) B) Kaplan-Meier overall survival according to tumor contact with the hippocampus (HPC). HR = hazard ratio.. NR = not reached (not calculatable)*.*

At time of censoring, 107 (78%) of the 138 patients within our cohort had deceased, with a median OS from start of RT of 8.4 months (range 0.23–64.7 months). In LUAD (73.9% of the population), we observed a median OS rate of 9.6 months and for LUSC 5.1 months, which were not significantly different (HR 1.23, 95% CI 0.71–2.16, p = 0.459, see Supplementary Fig. 2). Lesion contact with the SVZ was associated with significantly shorter OS (median 5.7 vs. 9.6 months; p = 0.019), as was contact with the HPC (median 3.2 months vs. 8.6 months; p = 0.0257), as shown in Fig. 3**.** Multivariable Cox regression adjusting for confounding variables confirmed that SVZ contact remained significantly associated with worse OS (HR 1.968 [95% CI 1.094–––3.542], p = 0.023). Similarly, HPC contact was still significantly associated with worse OS in multivariable Cox analysis (HR 5.751 [95% CI 1.733–19.07], p = 0.004) (see: Supplementary Table 1 and 2 for the detailed analyses). The adjusted VIFs for all variables and models (Supplementary Tables 3A − 3C, and 4A − 4C) were consistently low, with all values well below 2. This indicates that there is no significant multicollinearity among the predictors, ensuring the stability and reliability of the estimated coefficients.

### Survival analysis on subventricular zone and hippocampus dose

To address the potential concern that higher SVZ or HPC doses might simply reflect greater intracranial disease burden or more central lesion location, we first quantified how strongly radiotherapy dose to the neurogenic niches correlates with GTV/PTV size, number of BMs, and lesion centrality. This step was necessary to determine whether SVZ/HPC dose has an association with OS beyond its relationship with tumor burden and anatomical position. We found that increasing GTV and PTV volumes were both moderately associated with higher mean dose to the SVZ and hippocampi, and that patients with a higher number of brain metastases similarly tended to receive higher SVZ and hippocampal doses (Supplementary Fig. 3). In contrast, the correlation between PTV distance from the brain midpoint and SVZ dose was weak and non–significant, while PTVs located closer to the brain midpoint received somewhat higher hippocampal doses. For the distance calculation, we restricted the analysis to the 71 patients with a single brain metastasis, for whom the distance between the PTV center of gravity and the center of a template brain could be reliably defined.

Table 2, Table 3 show the results of the univariable- and multivariable Cox-regression between mean SVZ/HPC dose and OS, respectively. Both the unintended radiotherapy dose on SVZ and HPC have a significant negative association with OS after adjustment for covariates with HR 1.306 ([95% CI confidence interval (CI) 1.043–1.635], p = 0.019 and 1.222 ([95% CI 1.008–1.483], p = 0.041) per Gy, respectively. The consistently low adjusted VIF values, all falling between approximately 1.0 and 1.5 across all models and analyses, indicate a lack of significant multicollinearity among the predictors. This suggests that the independent variables in the models are not overly correlated with each other, allowing for reliable interpretation of their individual effects on survival. The adjusted VIFs for all variables and models are shown in Supplementary Tables 5A − 5C, and 6A − 6C**.**

**Table 2:** Univariable and multivariable Cox regression analysis of dose on the subventricular zone (SVZ) on overall survival (OS). Analyses with p < 0.05 are considered significant and bold in the table.

Univariable: (n = 138)		p-value HR		0.095 1.121 0.980 1.283
		95.0% CI	Lower	
			Upper	
Multivariable, corrected for: (n = 117)	Age, Sex, WHO performance status, type of lung cancer, SVZ volume	p-value		0.136
		HR		1.123
	
		95.0% CI	Lower	0.964
			Upper	1.308
Multivariable, corrected for: (n = 114)	Age, Sex, WHO performance status, type of lung cancer, SVZ volume, number of brain metastases, metastases elsewhere in the body, chemotherapy, immunotherapy, resection extent	p-value		**0.013**
		HR		**1.282**
		95.0% CI	Lower	**1.052**
			Upper	**1.561**
Multivariable, corrected for: (n = 114)	Age, Sex, WHO performance status, type of lung cancer, SVZ volume, number of brain metastases, metastases elsewhere in the body, chemotherapy, immunotherapy, resection extent, GTV volume	p-value		**0.019**
		HR		**1.306**
	
		95.0% CI	Lower	**1.043**
			Upper	**1.635**

GTV = Gross tumor volume, SVZ = subventricular zone, WHO performance status = World Health Organization performance status.

**Table 3:** Univariable and multivariable Cox regression analysis of dose on the hippocampus (HPC) on overall survival (OS). Analyses with p < 0.05 are considered significant and bold in the table.

Univariable: (n = 138)		p-value HR		0.205 1.090 0.955 1.267
		95.01% CI	Lower	
			Upper	
Multivariable, corrected for: (n = 117)	Age, Sex, WHO performance status, type of lung cancer, SGZ volume	p-value		0.248
		HR		1.091
	
		95.0% CI	Lower	0.941
			Upper	1.265
Multivariable, corrected for: (n = 114)	Age, Sex, WHO performance status, type of lung cancer, SGZ volume, number of brain metastases, metastases elsewhere in the body, chemotherapy, any other systemic therapy, resection extent	p-value		**0.020**
		HR		**1.227**
	
		95.0% CI	Lower	**1.031**
			Upper	**1.461**
Multivariable, corrected for: (n = 114)	Age, Sex, WHO performance status, type of lung cancer, SGZ volume, number of brain metastases, metastases elsewhere in the body, chemotherapy, any other systemic therapy, resection extent, GTV volume	p-value		**0.041**
		HR		**1.222**
		
		95.0% CI	Lower	**1.008**
			Upper	**1.483**

HPC = Hippocampus, GTV = Gross tumor volume, SGZ = subgranular zone, WHO performance status = World Health Organization performance status.

As part of a sensitivity analysis, we repeated the above calculations after removing 10% (n = 14) of the patients with the largest PTVs. None of the comparison remained significant after the removal of the subjects with the largest PTVs. Supplementary Tables 7 and 8 show the detailed results.

For exploration purposes, we repeated the SVZ/HPC dose-survival analyses only in patients with a single BM lesion. Arguably, this subgroup represents the most common SRS population with more limited intracranial disease, thereby reducing confounding from multiple lesions and extensive target volumes. Additionally, because the most central lesions (i.e. the 10% of patients with the shortest AC–PTV CoG distance) are more likely to unavoidably irradiate neurogenic niches, we also repeated the analyses after excluding this most central decile of lesions to test the robustness of our findings. In patients with a single BM lesion, the SVZ-based results remained significant across all model setups, with HRs of 1.51, 1.44, 1.67 and 1.64 with increasing model complexity, similarly as presented in Table 2. However, when the most centrally located tumors were excluded, only the univariable model remained significant. At the same time, none of the HPC-based Cox regressions yielded a significant relationship, regardless of patient selection or model setup. Notably, some of these exploratory analyses were performed in substantially smaller subgroups (n = 51–71), i.e. less than half the sample size of the main analyses in Table 2, Table 3. Detailed results are provided in Supplementary Table 9.

## Discussion

In this retrospective study, we investigated whether lesion contact with SVZ or HPC or unintended radiotherapy dose to this neurogenic niches is associated with OS of patients with BMs from NSCLC. Both lesion contact with the SVZ/HPC and unintended dose to these areas was significantly associated with worse OS. After adjusting for confounders, multivariable analysis showed a negative association between OS and unintended radiotherapy dose to the SVZ or the HPC, with a stronger effect for the SVZ. The current study adds significant value to the field of neuro-oncology by providing novel insights into the impact of unintended radiotherapy dose on the neurogenic niches in NSCLC patients with BMs. We used high-quality imaging data, novel MRI processing methods and a specially developed atlas [26] to enable accurate delineation of the SVZ region. While patients were recruited from a tertiary academic center, the cohort broadly reflects the characteristics of Dutch patients with brain metastases from NSCLC, thereby supporting the generalizability of our findings within this national healthcare landscape and suggesting that they may also be relevant to comparable patient populations internationally.

Consistent with our hypothesis, Kaplan-Meier analysis revealed lesion contact with the SVZ/HPC significantly associated with worse median OS (SVZ: 5.7 vs. 9.6 months, p = 0.019; HPC: 3.2 vs. 8.6 months, p = 0.026). This suggests that involvement of these neurogenic niches may promote metastatic tumor progression and disrupt brain tissue repair, contributing to a poorer prognosis. However, the small number of patients with HPC contact (n = 5) warns for caution and a potential bias, as similar patients with lesion-affected hippocampus may have died early or been too frail, possibly with cognitive decline, for RT, complicating interpretation of HPC-related survival outcomes. Multivariable Cox regression analysis confirmed SVZ contact as independent association of worse OS (HR 1.968 [95% CI 1.094–3.542], p = 0.023). Similarly, HPC contact remained significantly associated (HR 5.751 [95% CI 1.733–19.07], p = 0.004), though findings remain exploratory due to limited sample size. These results highlight neurogenic niche interactions as prognostic factors warranting validation in larger cohorts.

Our data further indicates that dose exposure to both the SVZ and HPC is partly driven by PTV, with larger PTVs resulting in a higher dose to the neurogenic niches. Interestingly, distance from the brain midpoint had minimal to no impact on niche doses. Both SVZ and HPC doses emerged as independent predictors of survival after adjusting for tumor burden. Sensitivity analyses confirmed SVZ dose remained significantly associated with OS even in single-lesion patients (HR 1.64 [95% CI 1.05–2.56], p = 0.029) and after excluding central lesions, while HPC associations were not significant anymore. These findings underscore SVZ's higher organ-at-risk priority over HPC for treatment planning, with implications for deliverability and dosimetry optimization in SRS, particularly for smaller lesions requiring disproportionate PTV expansion. These findings emphasize the importance of considering target volume effects when interpreting radiation dose associations with survival and neurocognitive outcomes.

The SVZ plays a pivotal role in adult neurogenesis and brain repair mechanisms [17], [45], [46], [47]. Therefore, it could be hypothesized that radiation-induced damage to the SVZ reduces brain repair capacity, thereby negatively impacting OS. In addition, previous research has highlighted the role of adult NSCs in the HPC in regulating cognitive functions such as learning and memory [21]. Interestingly, cognitive decline, independent of baseline cognition, has been linked to increased mortality, suggesting a potential indirect pathway between neurogenic damage and shortened OS [48]. However, our retrospective dataset lacks neurocognitive data to test this mechanism directly, which prospective studies such as our recently initiated ‘Cohort for patient-reported Outcomes, Imaging and trial inclusion in Metastatic BRAin disease’ (COIMBRA) (*ClinicalTrials.gov Identifier: NCT05267158)* are designed to address [49].

This insight has formed the basis of hippocampal-sparing radiotherapy. RCTs in other populations already have shown that HA-WBRT can significantly reduce cognitive decline [50]. Despite these findings, it is notable that the landmark trial of HA-WBRT in Small Cell Lung Cancer by Brown and colleagues [51], did not detect an OS benefit from hippocampal avoidance (median, 6.3 [HA-WBRT] vs. 7.6 [normal WBRT] months, respectively; HR 1.13 ([95% CI, 0.90–1.41], P = 0.31). This aligns with our finding of a relatively weaker HPC effect, most evident in single lesion patients where SVZ dose remained significant while HPC dose did not. It seems that a nuanced interdependence exists between the SVZ and HPC. The impact on OS may depend on the unintended dose received by both bilateral neurogenic niches and neglecting one could potentially obscure the observed association. Importantly, in our analysis the association was stronger for the SVZ (HR 1.306 [95% CI 1.043–1.635], p = 0.019 per Gy) that for the HPC (HR 1.222, [95% CI 1.008–1.483], p = 0.041 per Gy), suggesting a more substantial influence of the SVZ irradiation on OS. Currently, there is a limited number of investigations reporting the relationship between unintended dose received by the SVZ regions in CNS tumors and neurocognitive decline after radiotherapy, which showed deterioration in cognition after higher radiation dose to the SVZ [52]. More investigation is needed to explore the impact of radiation dose on the SVZ in CNS tumors and subsequent neurocognitive decline after radiotherapy. The ongoing prospective COIMBRA trial [49] exemplifies such an initiative which has the potential to answer this question.

Extracranial disease progression is the predominant cause of death in NSCLC patients with BMs rather than intracranial disease alone [53], [54]. While systemic progression-free survival data was unavailable due to decentralized care, baseline extracranial metastases were balanced across treatment groups and included in multivariable analyses, reducing confounding. In our region, UMC Utrecht is a tertiary referral center that provides specialized care, such as radiotherapy for metastatic brain lesions, which is not routinely available in other hospitals. As a result, many patients receive their brain-directed treatment at UMC Utrecht but continue their care for their primary disease and follow-up at the referring institutions. This fragmented care pathway creates substantial logistical barriers to accessing complete primary oncologic records and reliably determining the exact cause of death, which limits our ability to fully characterize systemic disease trajectories. Furthermore, only a minority of patients had lesions contacting SVZ or HPC (SVZ: 34/138; HPC: 5/138), consistent with the literature [19], [55]. The association between unintended radiation dose to these neurogenic niches and OS likely reflects indirect biological mechanisms, such as disruption of neural stem cell function or neurocognitive decline, independent of extracranial disease burden.

Our study faces several limitations. First, the imbalanced distribution between groups, those with lesions contacting the SVZ or HPC and those without (SVZ: 34 vs 104 and HPC: 5 vs 133) restricted our ability to perform subgroup analyses [19], [52]. While the inclusion of patients referred from multiple hospitals enhanced cohort diversity, it also resulted in missing data regarding specific gene mutations (e.g., EGFR, ALK, KRAS), progression free survival (PFS), follow-up, line of systemic therapy, and subsequent radiotherapy courses, preventing stratified survival analysis by these variables. Neurocognitive data was unavailable, since assessments were not routinely collected. Furthermore, referral bias may affect generalizability, as patients were recruited from a tertiary academic center and multiple referring hospitals, potentially selecting a clinically distinct cohort with lower rates of extracranial metastases due to prioritization of brain-radiotherapy. We recognize this as a key shortcoming and emphasize the necessity of prospective collection of clinical, neurocognitive and QoL-metrics in future research. Despite these limitations, the multi-center design strengthens internal validity and provides real-world insights applicable to similar referred patient populations.

This study shows the potential risks of unintended radiation dose to the neurogenic niches. This opens a new line of research in not only minimizing harm but potentially enhancing recovery or neuroprotection. However, identifying a biological or clinical principle alone is not sufficient to change practice. Any attempt to protect these regions must also be evaluated against practical constraints, particularly from a dosimetric and technical perspective. For example, it remains to be determined to what extent it is feasible to generate treatment plans that maintain adequate target coverage and conformity, respect existing dose constraints for established organs at risk, and simultaneously spare the SVZ and hippocampal complex. These trade–offs are likely to depend on lesion size, number, and location, as well as on the delivery technique and planning system, as has already been shown for hippocampal–avoidance WBRT with simultaneous integrated boost in several planning studies [56], [57], [58]. Dosimetric feasibility studies are therefore a necessary next step to quantify achievable dose reductions to the neurogenic niches under realistic clinical conditions, to identify patient subgroups in whom sparing is technically acceptable. Future research should explore whether targeted protection of neurogenic zones as putative organs at risk can be implemented in a dosimetrically feasible manner, and whether such strategies can ultimately shift the trajectory of both survival and cognitive outcomes in patients with BM.

In conclusion, our study shows a median OS of 8.3 months in selected patients with BMs from NSCLC. We found that both with lesion contact and any RT dose to the neurogenic niches, such as the SVZ and HPC, is correlated with poorer OS, consistent with previous observation in patients with glioma [26]. While primary gliomas are characterized by intracranial-dominant mortality and NSCLC brain metastases often involve extracranial-dominant progression (60–70% of deaths) [53], [54], the observed independent negative effects of neurogenic niche irradiation on OS across both tumor types suggest shared underlying biological mechanisms. These regions are crucial for brain repair and cognitive function, implying that their preservation could indirectly contribute to better quality of life and OS. Based on this data, we believe that avoidance of these niches is important to potentially extend the OS. When avoidance is not possible, the potential risks and benefits of RT strategies should be carefully weighed. To confirm our findings on OS and to better understand the effects of radiotherapy on neurocognitive function in patients with BMs, it is necessary to conduct larger, prospective trials with defined follow-up and standardized reporting.

## Waiver of patient consent

This is a retrospective case study. Patient consent has been waived by Ethic committee.

## Consent for publication

Not applicable.

## Funding

The authors declare that no funds, grants, or other support were received during the preparation of this manuscript.

## Declaration of competing interest

The authors declare that they have no known competing financial interests or personal relationships that could have appeared to influence the work reported in this paper.

## Data Availability

Due to patient privacy concerns, the raw data for this study cannot be shared.
