# Supplementary material for: Decreased overall survival in patients with brain metastases from non-small cell lung cancer with radiotherapy dose on the neurogenic niches
Source: Tech Innov Patient Support Radiat Oncol. 2026 Jan 27;37:100380. doi: 10.1016/j.tipsro.2026.100380 (PMC12886072; doi:10.1016/j.tipsro.2026.100380)
Supplement: Supplementary Data 1 [file mmc1.docx]

**SUPPLEMENTARY DATA**

| **Supplementary Text 1: Unix script** |
| --- |

#!/bin/bash

# Check if the correct number of arguments is provided

if [ "$#" -ne 3 ]; then

echo "Usage: bash dose_to_sfed.sh [input_dose_file] [current_number_of_fractions] [alpha_beta_ratio_for_conversion]"

echo "Example: bash dose_to_sfed.sh dose.nii.gz 3 2"

exit 1

fi

# Assign arguments to variables

input=$1

fractions=$2

alphabeta=$3

# Check if the input file exists

if [ ! -f "$input" ]; then

echo "Error: Input file $input does not exist"

exit 1

fi

# Extract the base name of the input file (without extension)

basename=$(basename "$input" .nii.gz)

echo "Processing: $input"

# Step 1: Calculate per-fraction dose map

fslmaths "$input" -div "$fractions" "${basename}_per_fraction"

# Step 2: Create voxelwise correction map

fslmaths "${basename}_per_fraction" -div "$alphabeta" -add 1 "${basename}_correction_map"

# Step 3: Calculate BED equivalent map

fslmaths "$input" -mul "${basename}_correction_map" "${basename}_BED"

# Step 4 & 5: Calculate SFED

fslmaths "${basename}_BED" -mul 4 -div "$alphabeta" -add 1 -sqrt -sub 1 -mul "$alphabeta" -div 2 "${basename}_SFED"

# Clean up intermediate files

rm "${basename}_per_fraction.nii.gz" "${basename}_correction_map.nii.gz" "${basename}_BED.nii.gz"

echo "Processing complete. SFED map created: ${basename}_SFED.nii.gz"


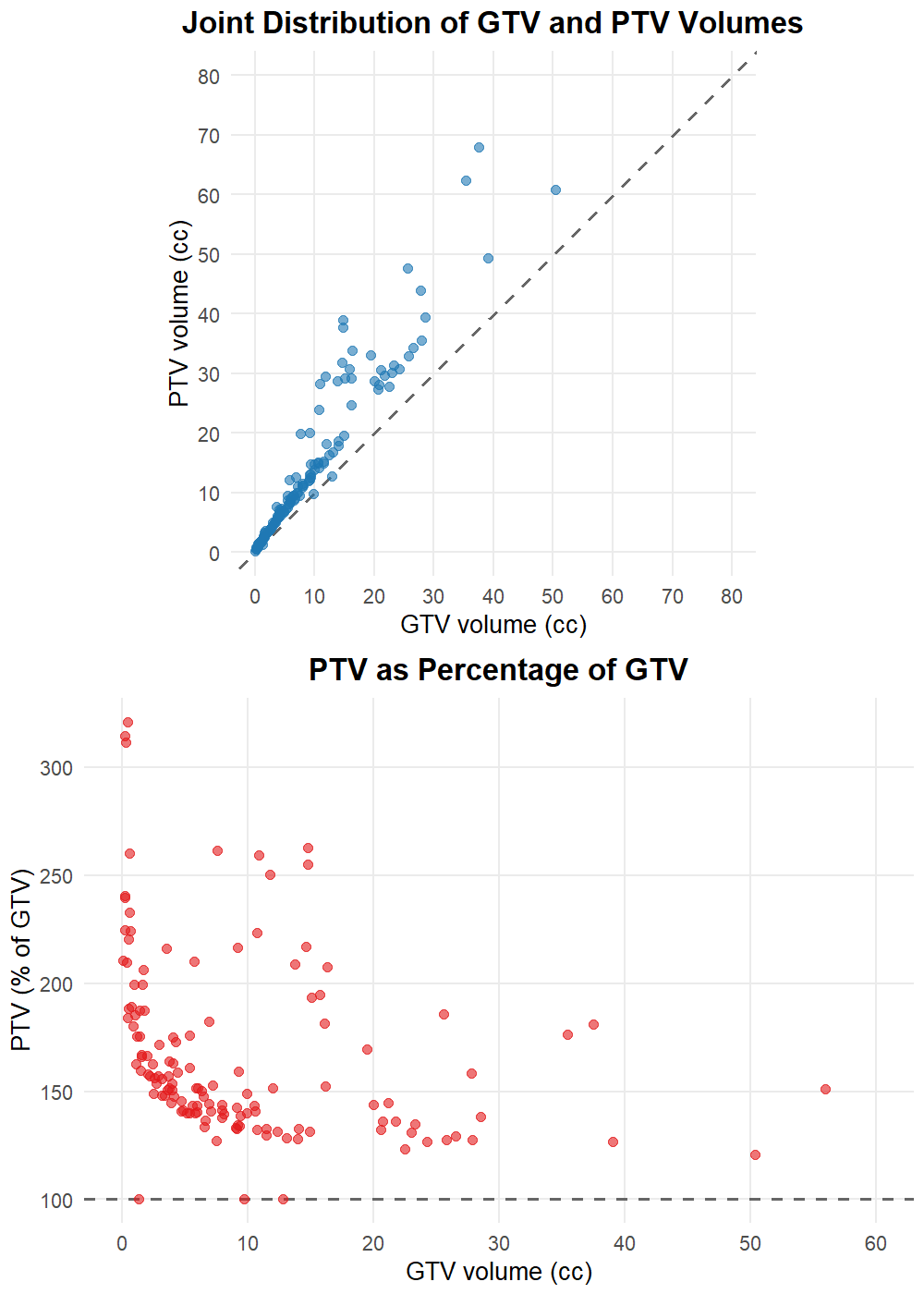


**Supplementary figure 1:** Top: Joint distribution of GTV and PTV volumes showing the absolute volumes in cubic centimeters. The dashed line represents the line of unity (PTV = GTV). Most data points lie above this line, indicating that PTV volumes consistently exceed GTV volumes due to clinical target volume margins and setup uncertainties. Bottom: PTV as percentage of GTV demonstrates the relative expansion from GTV to PTV as a function of GTV size. The dashed line at 100% represents unity. Smaller GTVs show proportionally larger PTV expansions (up to >300%), while larger GTVs approach more modest relative expansions. This inverse relationship reflects the fixed margin approach in treatment planning, where absolute margin sizes represent a larger relative proportion for smaller tumors.


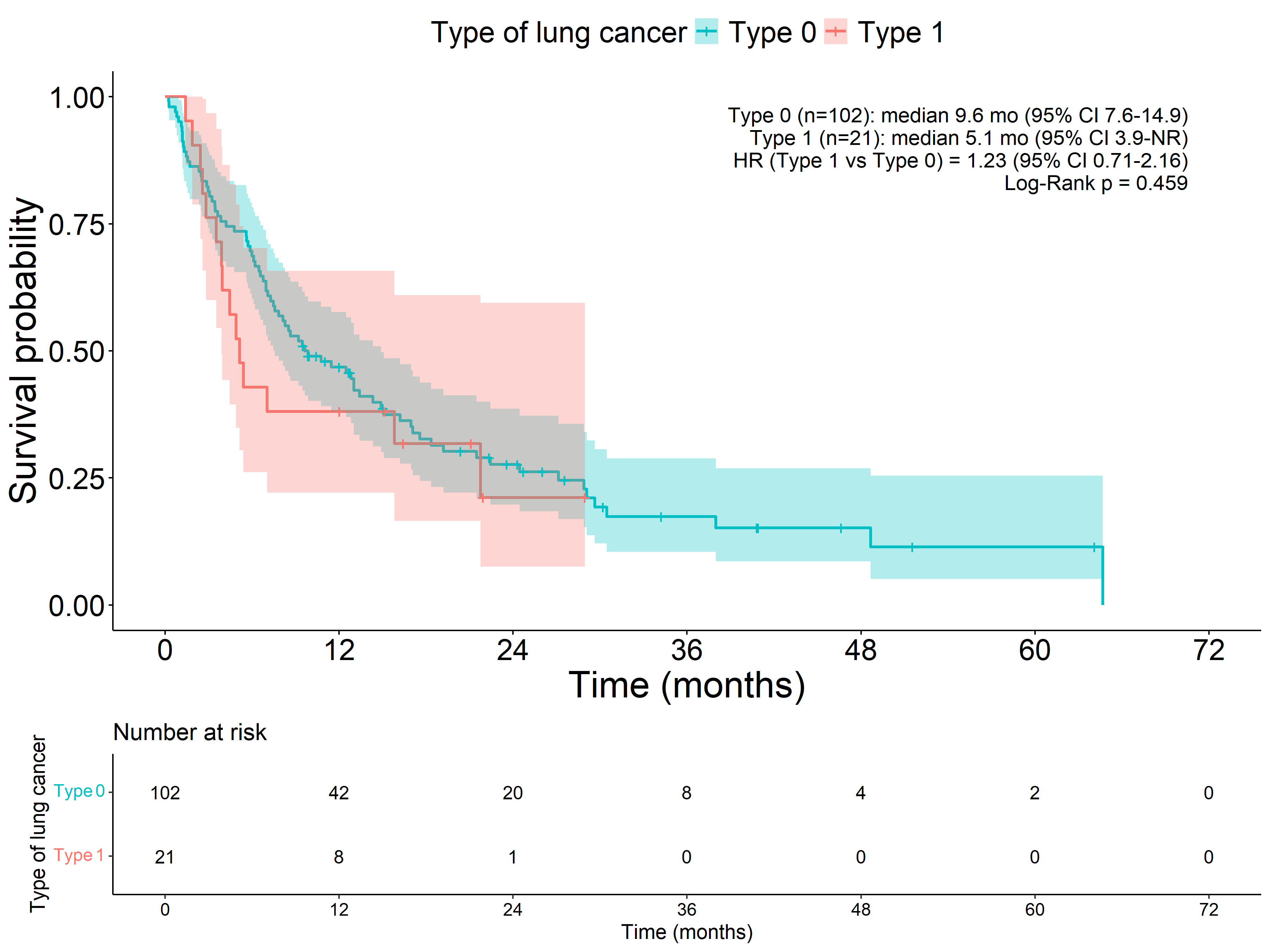


**Supplementary figure 2: Kaplan-Meier overall survival according lung cancer type. Type 0 = LUAD, while Type 1 = LUSC.** *HR=hazard ratio,LUAD=lung adenocarcinoma, LUSC=lung squamous cell carcinoma.*

| Supplementary table 1: Univariable and multivariable Cox regression analysis of no tumor contact/tumor contact with the subventricular zone (SVZ) on overall survival (OS). Analyses with p < 0.05 are considered significant and bolded in the table. | | | | | |
| --- | --- | --- | --- | --- | --- |
| Univariable: |  | p-value  HR | | | **0.002**  **1.668**  **1.083**  **2.569** |
|  |  | 95.0 % CI | | Lower |  |
|  |  |  |  | Upper |  |
| Multivariable, corrected for: | Age, Sex, WHO performance status, type of lung cancer, SVZ volume | p-value | | | **0.006**  **2.000** |
|  |  | HR | | |  |
|  |  |  | | | **1.222** |
|  |  | 95.0% CI | Lower | |  |
|  |  |  | Upper | | **3.274** |
| Multivariable, corrected for: | Age, Sex, WHO performance status, type of lung cancer, SVZ volume, number of brain metastases, metastases elsewhere in the body, chemotherapy, any other systemic therapy, resection extent | p-value | | | **0.014**  **1.995** |
|  |  | HR | | |  |
|  |  | 95.0% CI | Lower | | **1.147** |
|  |  |  | Upper | | **3.469** |
| Multivariable, corrected for: | Age, Sex, WHO performance status, type of lung cancer, SVZ volume, number of brain metastases, metastases elsewhere in the body, chemotherapy, any other systemic therapy, resection extent, GTV volume | p-value | | | **0.023** |
|  |  | HR | | | **1.968** |
|  |  | 95.0% CI | Lower | | **1.094** |
|  |  |  | Upper | | **3.542** |

| Supplementary table 2: Univariable and multivariable Cox regression analysis of no tumor contact/tumor contact with the hippocampus (HPC) on overall survival (OS). Analyses with p < 0.05 are considered significant and bolded in the table. | | | | |
| --- | --- | --- | --- | --- |
| Univariable: |  | p-value  HR | | **0.031**  **2.695**  **1.090**  **6.661** |
|  |  | 95.0% CI | Lower |  |
|  |  |  | Upper |  |
| Multivariable, corrected for: | Age, Sex, WHO performance status, type of lung cancer, SGZ volume | p-value  HR | | 0.133  2.168  0.788  5.965 |
|  |  |  | |  |
|  |  | 95.0 % CI | Lower |  |
|  |  |  | Upper |  |
| Multivariable, corrected for: | Age, Sex, WHO performance status, type of lung cancer, SGZ volume, number of brain metastases, metastases elsewhere in the body, chemotherapy, any other systemic therapy, resection extent | p-value | | **0.002** |
|  |  | HR | | **6.192** |
|  |  |  | |  |
|  |  | 95.0% CI | Lower | **1.908** |
|  |  |  | Upper | **20.09** |
| Multivariable, corrected for: | Age, Sex, WHO performance status, type of lung cancer, SGZ volume, number of brain metastases, metastases elsewhere in the body, chemotherapy, any other systemic therapy, resection extent, GTV volume | p-value |  | **0.004** |
|  |  | HR |  | **5.751** |
|  |  |  |  |  |
|  |  | 95.0% CI | Lower | **1.733** |
|  |  |  | Upper | **19.07** |

GTV = Gross tumor volume, SVZ = subventricular zone, WHO performance status = World Health Organization performance status

*HPC: Hippocampus, GTV = Gross tumor volume, SGZ= subgranular zone, WHO performance status = World Health Organization performance status*

**Supplementary table 3A - 3C: Adjusted Variance Inflation Factor (VIF) values for SVZ contact models**

Adjusted VIF was calculated as GVIF^(1/(2Df)) to account for degrees of freedom in categorical predictors. N indicates the number of patients included in each model. All VIF values below 5 indicate acceptable multicollinearity.  *cc = cubic centimeters, Df = degree of free, GTV = Gross Tumor Volume, Mets = metastases, SVZ = subventricular zone, VIF = Variance Inflation Factor, WHO* performance status *= World Health Organization* performance status

| **Supplementary table 3A: Adjusted VIF values for SVZ mean dose model 1** | |
| --- | --- |
| **Predictor** | **Adjusted VIF** |
| SVZ_contact | 1.111 |
| Age | 1.120 |
| Sex | 1.105 |
| WHO_performance_status | 1.028 |
| Type_lungcancer | 1.062 |
| SVZ_volume | 1.079 |

Model 1 includes SVZ contact, Age, Sex, WHO performance status, lung cancer type, and SVZ volume (N = 117).

| **Supplementary table 3B: Adjusted VIF values for SVZ mean dose model 2** | |
| --- | --- |
| **Predictor** | **Adjusted VIF** |
| SVZ_contact | 1.151 |
| Age | 1.104 |
| Sex | 1.090 |
| WHO_performance_status | 1.134 |
| Type_lungcancer | 1.109 |
| SVZ_volume | 1.064 |
| Mets_number | 1.129 |
| Extracranial_metastases | 1.102 |
| Chemotherapy | 1.140 |
| Immunotherapy_target_therapy | 1.091 |
| Extent_surgery | 1.064 |

Model 2 extends Model 1 with number of metastases, extracranial metastases, chemotherapy, immunotherapy/targeted therapy, and extent of surgery (N = 114).

| **Supplementary table 3C: Adjusted VIF values for SVZ mean dose model 3** | |
| --- | --- |
| **Predictor** | **Adjusted VIF** |
| SVZ_contact | 1.223 |
| Age | 1.132 |
| Sex | 1.092 |
| WHO_performance_status | 1.134 |
| Type_lungcancer | 1.111 |
| SVZ_volume | 1.065 |
| Mets_number | 1.133 |
| Extracranial_metastases | 1.121 |
| Chemotherapy | 1.143 |
| Immunotherapy_target_therapy | 1.119 |
| Extent_surgery | 1.071 |
| GTV_volume_cc | 1.138 |

Model 3 extends Model 2 with GTV volume in cc (N = 114).

**Supplementary table 4A - 4C: Adjusted Variance Inflation Factor (VIF) values for HPC contact models**

Adjusted VIF was calculated as GVIF^(1/(2Df)) to account for degrees of freedom in categorical predictors. N indicates the number of patients included in each model. All VIF values below 5 indicate acceptable multicollinearity. *cc = cubic centimeters, Df = degree of free, GTV = Gross Tumor Volume, HPC = hippocampus, Mets = metastases, SVZ = subventricular zone, VIF = Variance Inflation Factor, WHO* performance status *= World Health Organization* performance status

| **Supplementary table 4A: Adjusted VIF values for HPC mean dose model 1** | |
| --- | --- |
| **Predictor** | **Adjusted VIF** |
| HPC_contact | 1.213 |
| Age | 1.061 |
| Sex | 1.078 |
| WHO_performance_status | 1.018 |
| Type_lungcancer | 1.102 |
| SGZ_volume | 1.256 |

Model 1 includes HPC contact + Age, Sex, WHO, Type, SGZ_volume (N = 117)

| **Supplementary table 4B: Adjusted VIF values for HPC mean dose model 2** | |
| --- | --- |
| **Predictor** | **Adjusted VIF** |
| HPC_contact | 1.261 |
| Age | 1.080 |
| Sex | 1.083 |
| WHO_performance_status | 1.165 |
| Type_lungcancer | 1.147 |
| SGZ_volume | 1.181 |
| Mets_number | 1.224 |
| Extracranial_metastases | 1.133 |
| Chemotherapy | 1.177 |
| Immunotherapy_target_therapy | 1.104 |
| Extent_surgery | 1.089 |

Model 2 extends Model 1 with number of metastases, extracranial metastases, chemotherapy, immunotherapy/targeted therapy, and extent of surgery (N = 114).

| **Supplementary table 4C: Adjusted VIF values for HPC mean dose model 3** | |
| --- | --- |
| **Predictor** | **Adjusted VIF** |
| HPC_contact | 1.286 |
| Age | 1.087 |
| Sex | 1.086 |
| WHO_performance_status | 1.170 |
| Type_lungcancer | 1.156 |
| SGZ_volume | 1.187 |
| Mets_number | 1.224 |
| Extracranial_metastases | 1.148 |
| Chemotherapy | 1.181 |
| Immunotherapy_target_therapy | 1.113 |
| Extent_surgery | 1.096 |
| GTV_volume_cc | 1.061 |

Model 3 extends Model 2 with GTV volume in cc (N = 114).


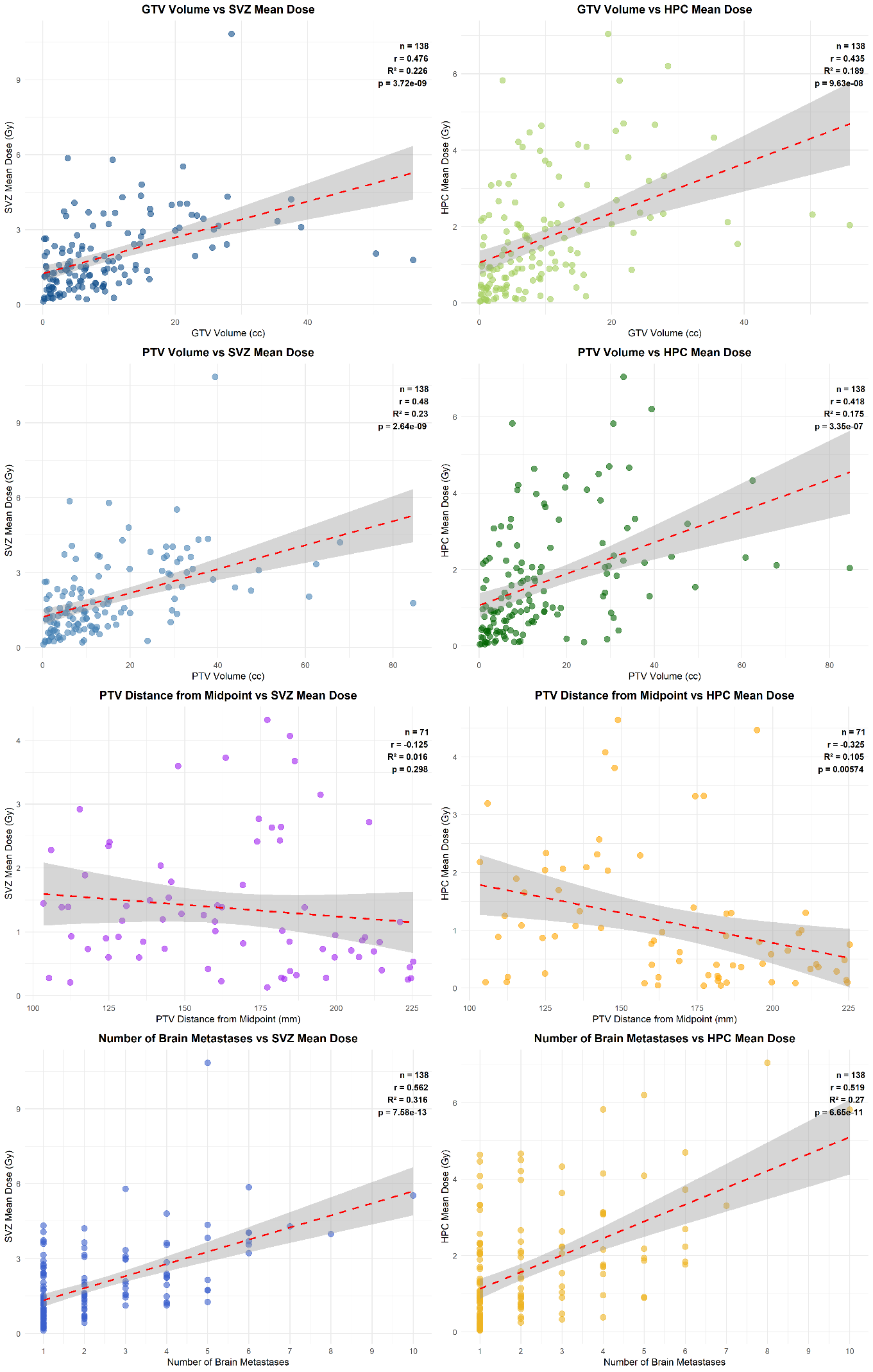


**Supplementary figure 3:** Relationship between intracranial disease burden, lesion centrality, and mean dose to the neurogenic niches. Top row: Scatterplots show that increasing GTV volume is associated with higher mean dose to both the SVZ (left) and hippocampi (right). Second row: Larger PTVs similarly receive higher mean SVZ (left) and hippocampal (right) doses. Third row: In the subgroup of patients with a single brain metastasis (n = 71), plots display the association between lesion centrality, approximated by the distance from the PTV center of gravity to the brain midpoint, and mean SVZ (left) and hippocampal (right) dose. SVZ dose shows little dependence on lesion centrality, whereas more centrally located PTVs are associated with somewhat higher hippocampal doses. Bottom row: Scatterplots indicate that patients with a higher number of brain metastases tend to receive higher mean doses to both the SVZ (left) and hippocampi (right). Red dashed lines indicate linear regression fits, with grey bands representing 95% confidence intervals.

**Supplementary table 5A - 5C: Adjusted Variance Inflation Factor (VIF) values for SVZ mean dose models**

Adjusted VIF was calculated as GVIF^(1/(2Df)) to account for degrees of freedom in categorical predictors. N indicates the number of patients included in each model. All VIF values below 5 indicate acceptable multicollinearity.  *cc = cubic centimeters, Df = degree of free GTV = Gross Tumor Volume, Mets = metastases, SVZ = subventricular zone, VIF = Variance Inflation Factor, WHO* performance status *= World Health Organization* performance status.

| **Supplementary table 5A: Adjusted VIF values for SVZ mean dose model 1** | |
| --- | --- |
| **Predictor** | **Adjusted VIF** |
| SVZ_meanDose | 1.022 |
| Age | 1.044 |
| Sex | 1.045 |
| WHO_performance_status | 1.017 |
| Type_lungcancer | 1.032 |
| SVZ_volume | 1.034 |

Model 1 includes SVZ mean dose, Age, Sex, WHO performance status, lung cancer type, and SVZ volume (N = 117).

| **Supplementary table 5B: Adjusted VIF values for SVZ mean dose model 2** | |
| --- | --- |
| **Predictor** | **Adjusted VIF** |
| SVZ_meanDose | 1.252 |
| Age | 1.097 |
| Sex | 1.067 |
| WHO_performance_status | 1.128 |
| Type_lungcancer | 1.137 |
| SVZ_volume | 1.030 |
| Mets_number | 1.317 |
| Extracranial_metastases | 1.115 |
| Chemotherapy | 1.146 |
| Immunotherapy/target therapy | 1.086 |
| Extent_surgery | 1.050 |

Model 2 extends Model 1 with number of metastases, extracranial metastases, chemotherapy, immunotherapy/targeted therapy, and extent of surgery (N = 114).

| **Supplementary table 5C: Adjusted VIF values for SVZ mean dose model 3** | |
| --- | --- |
| **Predictor** | **Adjusted VIF** |
| SVZ_meanDose | 1.440 |
| Age | 1.112 |
| Sex | 1.066 |
| WHO_performance_status | 1.127 |
| Type_lungcancer | 1.136 |
| SVZ_volume | 1.030 |
| Mets_number | 1.332 |
| Extracranial_metastases | 1.122 |
| Chemotherapy | 1.148 |
| Immunotherapy/target therapy | 1.108 |
| Extent_surgery | 1.053 |
| GTV_volume_cc | 1.233 |

Model 3 extends Model 2 with GTV volume in cc (N = 114).

**Supplementary table 6A - 6C: Adjusted Variance Inflation Factor (VIF) values for HPC mean dose models**

Adjusted VIF was calculated as GVIF^(1/(2Df)) to account for degrees of freedom in categorical predictors. N indicates the number of patients included in each model. All VIF values below 5 indicate acceptable multicollinearity. *cc = cubic centimeters, Df = degree of free, GTV = Gross Tumor Volume, HPC = hippocampus, Mets = metastases, SVZ = subventricular zone, VIF = Variance Inflation Factor, WHO = World Health Organization*

| **Supplementary table 6A: Adjusted VIF values for HPC mean dose model 1** | |
| --- | --- |
| **Predictor** | **Adjusted VIF** |
| HPC_meanDose | 1.064 |
| Age | 1.042 |
| Sex | 1.048 |
| WHO_performance_status | 1.021 |
| Type_lungcancer | 1.063 |
| SGZ_volume | 1.051 |

Model 1 includes HPC mean dose + Age, Sex, WHO, Type, SGZ_volume (N = 117)

| **Supplementary table 6B: Adjusted VIF values for HPC mean dose model 2** | |
| --- | --- |
| **Predictor** | **Adjusted VIF** |
| HPC_meanDose | 1.255 |
| Age | 1.090 |
| Sex | 1.074 |
| WHO_performance_status | 1.152 |
| Type_lungcancer | 1.134 |
| SGZ_volume | 1.104 |
| Mets_number | 1.305 |
| Extracranial_metastases | 1.138 |
| Chemotherapy | 1.134 |
| Immunotherapy/target therapy | 1.092 |
| Extent_surgery | 1.055 |

Model 2 extends Model 1 with number of metastases, extracranial metastases, chemotherapy, immunotherapy/targeted therapy, and extent of surgery (N = 114).

| **Supplementary table 6C: Adjusted VIF values for HPC mean dose model 3** | |
| --- | --- |
| **Predictor** | **Adjusted VIF** |
| HPC_meanDose | 1.392 |
| Age | 1.110 |
| Sex | 1.076 |
| WHO_performance_status | 1.152 |
| Type_lungcancer | 1.153 |
| SGZ_volume | 1.106 |
| Mets_number | 1.317 |
| Extracranial_metastases | 1.140 |
| Chemotherapy | 1.135 |
| Immunotherapy/target therapy | 1.105 |
| Extent_surgery | 1.062 |
| GTV_volume_cc | 1.157 |

Model 3 extends Model 2 with GTV volume in cc (N = 114).

| Supplementary table 7: Univariable and multivariable Cox regression analysis of dose on the subventricular zone (SVZ) on overall survival (OS) without the top 10% of patients with the largest PTV. Analyses with p < 0.05 are considered significant and bolded in the table. | | | | |
| --- | --- | --- | --- | --- |
| Univariable:  (n = 124) |  | p-value  HR | | 0.228  1.102  0.940  1.29 |
|  |  | 95.0% CI | Lower |  |
|  |  |  | Upper |  |
| Multivariable, corrected for: (n = 105) | Age, Sex, WHO performance status, type of lung cancer, SVZ volume | p-value | | 0.623 |
|  |  | HR | | 1.048 |
|  |  |  | |  |
|  |  | 95.0% CI | Lower | 0.87 |
|  |  |  | Upper | 1.26 |
| Multivariable, corrected for: (n = 102) | Age, Sex, WHO performance status, type of lung cancer, SVZ volume, number of brain metastases, metastases elsewhere in the body, chemotherapy, immunotherapy, resection extent | p-value | | 0.204 |
|  |  | HR | | 1.176 |
|  |  | 95.0% CI | Lower | 0.92 |
|  |  |  | Upper | 1.51 |
| Multivariable, corrected for: | Age, Sex, WHO performance status, type of lung cancer, SVZ volume, number of brain metastases, metastases elsewhere in the body, chemotherapy, immunotherapy, resection extent, GTV volume | p-value | | 0.277 |
|  |  | HR | | 1.185 |
|  |  |  | |  |
|  |  | 95.0% CI | Lower | 0.87 |
|  |  |  | Upper | 1.61 |

| Supplementary table 8: Univariable and multivariable Cox regression analysis of dose on the hippocampus (HPC) on overall survival (OS) without the top 10% of patients with the largest PTV. Analyses with p < 0.05 are considered significant and bolded in the table. | | | | |
| --- | --- | --- | --- | --- |
| Univariable:  (n = 124) |  | p-value  HR | | 0.467  1.058  0.91  1.23 |
|  |  | 95.01% CI | Lower |  |
|  |  |  | Upper |  |
| Multivariable, corrected for: (n = 105) | Age, Sex, WHO performance status, type of lung cancer, SGZ volume | p-value | | 0.529 |
|  |  | HR | | 1.059 |
|  |  |  | |  |
|  |  | 95.0% CI | Lower | 0.89 |
|  |  |  | Upper | 1.26 |
| Multivariable, corrected for: (n = 102) | Age, Sex, WHO performance status, type of lung cancer, SGZ volume, number of brain metastases, metastases elsewhere in the body, chemotherapy, any other systemic therapy, resection extent | p-value | | **0.096** |
|  |  | HR | | **1.198** |
|  |  |  | |  |
|  |  | 95.0% CI | Lower | **0.97** |
|  |  |  | Upper | **1.48** |
| Multivariable, corrected for:  (n = 102) | Age, Sex, WHO performance status, type of lung cancer, SGZ volume, number of brain metastases, metastases elsewhere in the body, chemotherapy, any other systemic therapy, resection extent, GTV volume | p-value |  | **0.151** |
|  |  | HR |  | **1.179** |
|  |  |  |  |  |
|  |  | 95.0% CI | Lower | **0.94** |
|  |  |  | Upper | **1.48** |

**Supplementary table 9**: Univariable and multivariable Cox regression analysis of dose on the subventricular zone (SVZ) on overall survival (OS) of patients with a single BM lesion with and without the most centrally located lesion. Analyses with p < 0.05 are considered significant and bolded in the table. Multivariable model setup is identical as shown in Suppl. table 1.

| Dataset | Preditor | Model | N | HR | CI | p |
| --- | --- | --- | --- | --- | --- | --- |
| Single lesion only | SVZ_meanDose | Univariable | 71 | 1.51 | 1.16-1.95 | **0.002** |
|  |  | Multivariable Model1 | 62 | 1.44 | 1.05-1.97 | **0.023** |
|  |  | Multivariable Model2 | 60 | 1.67 | 1.08-2.59 | **0.022** |
|  |  | Multivariable Model3 | 60 | 1.64 | 1.05-2.56 | **0.029** |
|  | HPC_meanDose | Univariable | 71 | 1.22 | 0.98-1.53 | 0.079 |
|  |  | Multivariable Model1 | 62 | 1.17 | 0.91-1.51 | 0.229 |
|  |  | Multivariable Model2 | 60 | 1.26 | 0.93-1.7 | 0.135 |
|  |  | Multivariable Model3 | 60 | 1.24 | 0.89-1.71 | 0.198 |
| Single lesion only, 10 most central patients removed | SVZ_meanDose | Univariable | 61 | 1.51 | 1.15-1.98 | **0.003** |
|  |  | Multivariable Model1 | 53 | 1.35 | 0.97-1.87 | 0.071 |
|  |  | Multivariable Model2 | 51 | 1.46 | 0.93-2.31 | 0.104 |
|  |  | Multivariable Model3 | 51 | 1.4 | 0.88-2.22 | 0.152 |
|  | HPC_meanDose | Univariable | 61 | 1.22 | 0.96-1.55 | 0.097 |
|  |  | Multivariable Model1 | 53 | 1.16 | 0.87-1.54 | 0.31 |
|  |  | Multivariable Model2 | 51 | 1.17 | 0.84-1.62 | 0.358 |
|  |  | Multivariable Model3 | 51 | 1.24 | 0.88-1.73 | 0.219 |
